# Bladder and Bowel Management in Adolescents and Young Adults with Multiple Sclerosis Since Childhood: Is Bowel Management Overlooked? A Case Series

**DOI:** 10.3390/children13020225

**Published:** 2026-02-05

**Authors:** Maria Laura Sollini, Chiara Pellegrino, Rebecca Pulvirenti, Maria Luisa Capitanucci, Antonio Maria Zaccara, Gabriella Lentini, Martina Monti, Gessica Della Bella, Massimiliano Valeriani, Giovanni Mosiello

**Affiliations:** 1 Division of Neuro-Urology, Bambino Gesù Children’s Hospital, Scientific Institute for Research, Hospitalization and Healthcar (IRCCS), 00165 Rome, Italy; marialaura.sollini@alumni.uniroma2.eu (M.L.S.); mluisa.capitanucci@opbg.net (M.L.C.); antoniomaria.zaccara@opbg.net (A.M.Z.); gabriellalentini98@gmail.com (G.L.); martinamonti3194@gmail.com (M.M.); giovanni.mosiello@opbg.net (G.M.); 2 Clinical Science and Translational Medicine, Tissue Engineering and Remodeling Biotechnologies for Body Function PhD School, University of Rome Tor Vergata, Via Cracovia 50, 00133 Rome, Italy; 3 Pediatric Surgery, Department of Neuroscience, Rehabilitation, Ophthalmology, Genetics, and Maternal-Child Sciences (DINOGMI), University of Genoa, Largo Paolo Daneo 3, 16132 Genoa, Italy; 4 Neurorehabilitation and Adapted Physical Activity Day Hospital, Bambino Gesù Children’s Hospital, Scientific Institute for Research, Hospitalization and Healthcar (IRCCS), 00165 Rome, Italy; gessica.dellabella@opbg.net; 5 Developmental Neurology Unit, Bambino Gesù Children’s Hospital, Scientific Institute for Research, Hospitalization and Healthcar (IRCCS), 00165 Rome, Italy; massimiliano.valeriani@opbg.net

**Keywords:** multiple sclerosis, bowel dysfunction, fecal, neurogenic bowel, bowel management, quality of life

## Abstract

**Highlights:**

**What are the main findings?**

• In our cohort of adolescent/young adult patients with multiple sclerosis since childhood and urological manifestations, 66% manifested bowel symptoms (predominantly constipation).
• A specific bowel management questionnaire showed an overall fair management, with a quality of life score poorer among those patients non-adherent to therapy.

**What are the implications of the main findings?**

• Early identification of bowel dysfunction using non-invasive methods, such as questionnaires, is essential to optimize bowel management.
• Early diagnosis and treatment can allow for less invasive therapy, increasing adherence to bowel management, and could allow for a better quality of life.

**Abstract:**

Background/Objectives: Multiple sclerosis (MS) is a chronic, demyelinating, inflammatory, and degenerative disease of the nervous system that may present in childhood or adolescence, defined as Pediatric-Onset Multiple Sclerosis (POMS). Among its diverse clinical manifestations, neurogenic bowel dysfunction (NBD), including chronic constipation and fecal incontinence, represents a distressing condition with a substantial impact on quality of life (QoL). Despite its clinical relevance, the evidence on bowel dysfunction in pediatric MS remains limited. The objective of this case series was to evaluate bowel dysfunction and its management in a pediatric MS cohort, and to assess whether it is associated with QoL. Methods: We reported the data on the urological and bowel conditions and on the quality of life of our pediatric patients affected by MS and urological dysfunction. We considered MS patients with urinary symptoms who were referred to our department between January 2019 and January 2024, only including individuals with symptoms onset before 18 years of age. In our series, the demographic and clinical data were reviewed, and patients were re-evaluated via telephone interview at least one year after initiation of treatment. The International Consultation on International Consultation on Incontinence Questionnaire-Urinary Incontinence Short Form (ICIQ-UI SF), Pediatric Bowel Management Scoring Tool (PBMST), and Pediatric Quality of Life Inventory (PedsQL, young adults’ version) were used to assess urinary and bowel function and related QoL outcomes. Results: Six patients (four females, two males; median age 14.5 years) were included. Bowel symptoms were reported in four cases (66.6%), predominantly constipation. The PBMST scores indicated overall fair bowel management, while the mean PedsQL score was 62.8/100, with poorer scores observed for the non-adherent patients. Conclusions: Bowel dysfunction constitutes an underrecognized but clinically significant manifestation in pediatric MS. Its early identification and adherence to a structured bowel management program seems to be crucial for optimizing symptom control and preserving quality of life. Standardized questionnaires represent effective, non-invasive tools for assessment and longitudinal monitoring.

## 1. Introduction

Multiple sclerosis (MS) is a chronic, inflammatory, demyelinating, and degenerative disease that affects the central nervous system (CNS), with a likely autoimmune pathogenesis [1]. MS represents a leading cause of disability in young individuals and has a profoundly negative impact on their quality of life (QoL), as well as that of their families and caregivers.

Pediatric forms of MS are defined as EOMS (Early-Onset MS), POMS (Pediatric-Onset Multiple Sclerosis) or juvenile MS [2,3,4], with an apparently increasing prevalence. The incidence of pediatric MS is 0.13–0.66 cases per 100,000 children annually. Approximately 10% of all MS patients experience their first demyelinating event before the age of 18 [3,4,5]. Among the various manifestations of multiple sclerosis, urinary and fecal symptoms may have a negative association with the quality of life of these delicate patients, in addition to neurological problems. At MS diagnosis, 10% of patients manifest lower urinary tract dysfunctions (LUTDs); after 10 years of disease, up to 80% complain about LUTDs, with a possible high impact on their quality of life and on renal function [5,6].

On the other hand, closely linked to the presence of urological symptoms is the appearance of intestinal symptoms, such as neurogenic bowel dysfunction (NBD). NBD can manifest as chronic constipation and fecal incontinence, sometimes coexisting together as “overflow diarrhea” due to solid stool impacting over the rectum, allowing only watery stool to pass [7]. Neurogenic bowel dysfunction in MS results from demyelination and axonal damage affecting the supraspinal, spinal, and autonomic pathways involved in gastrointestinal (GI) motor control [8]. Lesions in the corticospinal tracts, brainstem nuclei, and sacral spinal cord disrupt the coordination between the enteric nervous system and central autonomic regulation [7].

Some studies have demonstrated that MS patients frequently exhibit delayed gastric emptying, slow colonic transit, and abnormalities on anorectal manometry, indicating dysfunction of both upper and lower GI motility under neurological influence [9]. These findings suggest that demyelinating lesions affecting autonomic and somatic neural pathways can impair coordinated GI motor activity. NBD in MS involves impaired rectal sensation and anal sphincter dysfunction [8], which contribute to the onset of fecal symptoms.

A study by Levinthal suggests that approximately 65.6% of patients report GI symptoms, such as constipation (36.6%), dysphagia (21.1%), fecal incontinence (15.1%), and dyspeptic symptoms (~28.4%). Functional bowel syndromes are also described, such as functional dysphagia (14.7%), functional dyspepsia (16.5%), functional constipation (31.7%), and irritable bowel syndrome (19.3%) [10].

Chronic constipation and fecal incontinence can have a greater impact on QoL than urinary retention or incontinence because of the smell, which can lead to social isolation [11].

Unfortunately, to date, the literature on this subject is still scarce, especially in the pediatric population, with the few existing studies mainly concerning the urological aspect, thus making the management of NBD even less standardized [12].

Since neurogenic bladder and bowel conditions tend to coincide with complex clinical needs of these chronic patients, we decided to analyze, through simple questionnaires, patients’ perceptions of their urological and intestinal situations, and how these affect their quality of life. To achieve this aim, we reported on a cohort of MS patients referred to our Neuro-Urology Division with onset of urinary and/or bowel symptoms before the age of 18 years, who were admitted to our department from January 2019 to January 2024, with a minimum follow-up of 5 years. Within this cohort of patients, we evaluated their bowel management and examined how their bowel and bladder manifestations were associated with their quality of life.

## 2. Case Presentation

An MS diagnosis was determined according to the Krupp criteria [13]. A retrospective collection of demographic and clinical data, including onset of symptoms, magnetic resonance (MR) localization of demyelinating lesions, and urological manifestations and their management were conducted [14]. Then, we administered three questionnaires to all patients regarding the fecal and urological aspects and quality of life in relation to their clinical pattern:

- International Consultation on Incontinence Questionnaire-Urinary Incontinence Short Form (ICIQ-UI SF): As the patients had previously been studied for urological dysfunction, we administered this questionnaire to study the progression of the condition [15]. The total score is calculated by adding the scores for items 3, 4, and 5; the minimum score is 0 and the maximum is 21.
- The PBMST is a recently developed questionnaire designed to assess bowel function in children from the age of four years old. It includes a parent-proxy version for children aged 4 to 8 years, and a self-reported version for patients aged 8 years and older. It is usually utilized by our Institute for the valuation of bowel function, even in young adults. This dual structure enables the direct evaluation of symptoms while reducing reliance on caregiver impressions. The psychometric testing demonstrated strong internal consistency, good test–retest reliability, and satisfactory construct validity [16]. The Pediatric Bowel Management Scoring Tool (PBMST) was administered to obtain an objective parameter for assessing intestinal problems [16]. The score is calculated by adding the points for the various items and ranges from 0 to 22, and is then evaluated according to the score bands (as explained in Table 1).
- The Pediatric Quality of Life (PedsQL) questionnaire, young adults’ version, evaluates the impact of their symptoms on their daily life [17]. The total score corresponds to the converted sum of the scores for the individual items, up to a maximum of 100, where the highest score corresponds to a better quality of life. The PedsQL questionnaire investigates both the ability to perform actions, such as playing sports, and the social situations to which a patient is exposed and the emotions he/she experiences in everyday life. It provides us with a subjective assessment of the perception of quality of life.

**Table 1:** Description of urological and intestinal status.

Pt No; Sex	LUTSs	Urological FU	ICIQ-UI-SF Score (0–21)	Bowel Status	Bowel Management	PBMST
1; F	Urge incontinence; enuresis	Rejection of CIC; incontinence	19	Constipation	Rejection of therapy	2
2; F	Urinary retention; few episodes of urge incontinence	CIC; spontaneous micturition; recurrent cystitis	12	Constipation	Diet; TAI once/week	2
3; M	Incontinence	Rejection of CIC; incontinence	21	Constipation; soiling once/week	Diet; osmotic laxatives; TAI	6
4; F	Incontinence	Resolution of symptoms	0	No symptoms	No therapy	1
5; F	Incontinence	Resolution of symptoms	0	Constipation	Rejection of therapy	8
6; M	Urge incontinence; urgency	Resolution of symptoms	0	No symptoms	No therapy	0

Pt no: patient number; F: female; M: male; LUTSs: lower urinary tract symptoms; FU: follow-up; CIC: clean intermittent catheterization; TAI: transanal irrigation; PBMST: Pediatric Bowel Management Scoring Tool. PBMST score of 0–5 indicates fair bowel management; 6–7, bowel management is moderately valid; 8–10 is poor; up to 11 is considered very poor.

The strategies and concerns regarding bowel management (diet, fluid intake, laxatives, transanal irrigation (TAI), etc.) were evaluated.

All the questionnaires and interviews were conducted by the same clinician. Within 7 days, the questionnaires were administered to all patients, without standardizing the timing of the follow-up for each patient.

Written consent was obtained from the parents/caregivers/patients, as established by our institution’s guidelines.

### 2.1. Case 1

An 18.2-year-old female patient diagnosed with RR multiple sclerosis at 13.1 years of age, with concomitant onset of urge incontinence and enuresis, and a urodynamic diagnosis of detrusor overactivity. The urological management included CIC, anticholinergic therapy (oxybutynin), biofeedback for pelvic floor rehabilitation, and, subsequently, intradetrusor injection of Onabotulinum Toxin-A.

Regarding NBD, severe constipation was present; osmotic laxatives (polyethylene glycol 3350) and then TAI were prescribed.

At the last follow-up, the patient had discontinued CIC and NBD therapy, preferring to use continence devices (diapers).

### 2.2. Case 2

A 20.6-year-old female patient with RR-MS diagnosis at 15.6 years, and has a history of urinary retention managed with CIC. A few episodes of urge incontinence occurred approximately 16 months before the last urological evaluation and were treated with anticholinergics; no more episodes were recorded in the last year. Currently, she rarely experiences urinary hesitancy or retention, which is resolved with catheterization on demand.

She has a history of constipation, managed with diet and osmotic laxatives (polyethylene glycol 3350). Due to frequent episodes of fecal impaction in the past years, TAI has been administered.

At the last follow-up, she was no longer receiving therapy with laxatives, had normal stools, and is undergoing TAI 1–3 times/week, with good bowel control.

### 2.3. Case 3

A 25.3-year-old male patient diagnosed with RR-MS (age at diagnosis 17.3 years), an anorectal malformation (initially managed at another institute and then referred to our hospital), myelomeningocele (surgically corrected at birth at another hospital), and subsequent secondary tethered cord (treated at our center).

His neurogenic bladder has been managed with CIC since birth. Subsequently, oxybutynin and then intradetrusor injection of Onabotulinum Toxin-A have been added to the therapy. One year before his MS diagnosis he showed a worsening of his LUTSs (lower urinary tract symptoms), certified by a urodynamic study. For this reason, we increased frequency of CIC and Onabotulinum Toxin-A injections (every 6 months), without any benefits. With the suspicion of a re-tethering, we requested an MRI that excluded the low conus. Due to the progressive worsening of his LUTSs and the reduction in sensitivity in his lower limbs, a gadolinium contrast dye MRI highlighted lesions due to MS. Then, he progressively showed a reduction in adherence to all treatments, including urological management. At the last follow-up, he refused any urological therapy and the possibility of transitional care to an adult urology center, despite reporting that the presence of continuous urinary incontinence interfered with his daily life. He was assigned the maximum score for the relevant question on the ICIQ-UI-SF.

His neurogenic bowel dysfunction, with severe constipation, was originally managed with enemas in addition to osmotic laxatives (polyethylene glycol 3350), with poor results. He has a history of frequent episodes of fecal impaction and fecal incontinence. An anorectal manometry revealed: good rectal sensitivity threshold; adequate relaxation of the levator ani muscles during the straining maneuver; and poor voluntary contraction tone. The patient reported the use of an anal plug, which was poorly tolerated and then suspended.

Due to persistent difficulty with proper bowel emptying, TAI was successfully prescribed. At the last follow-up, the patient had no further fecal impaction, was taking osmotic laxatives (polyethylene glycol 3350), and performing TAI. He reported occasionally minimal soiling, mainly related to reduced TAI frequency.

### 2.4. Case 4

A 22.8-year-old female with RR-MS (diagnosed at 13.1 years of age), celiac disease, and difficulties with attention and anxious states, had a urological evaluation due to daily urine leakage, with an onset approximately 3 years after the MS diagnosis. She was initially treated with oxybutynin and then with percutaneous tibial nerve stimulation (PTNS). At the last follow-up, she revealed no more urinary symptoms.

Regarding her NBD, initially constipation was reported, which was easily managed with conservative management (diet, fluid intake, etc.).

She did not complain about any issues with constipation or fecal incontinence.

### 2.5. Case 5

A 21.8-year-old female patient with RR-MS diagnosed at 16.8 years was admitted for urinary leakage (began 1 year after MS diagnosis), which disappeared with MS treatment.

Persistent constipation, with thicker stools every 48–72 h was reported; osmotic laxative and then TAI were proposed. The patient shows very low adherence to the NBD treatment.

### 2.6. Case 6

A 20.4-year-old male with RR-MS diagnosis at 11.4 years had urological symptoms onset 5 years after the neurological diagnosis, with urgency and occasional urge incontinence. Timed voiding and anticholinergics easily solved the urological symptoms. His NBD constipation was well treated with conservative management (diet, fluid intake, etc.).

### 2.7. Urological Dysfunctions

#### 2.7.1. Urinary Status

Urinary symptoms were already present at the time of multiple sclerosis diagnosis in two patients, while in four, the symptoms appeared between 6 months and 5 years after the MS diagnosis.

The prevalent urinary symptoms were urinary incontinence in five patients (one also had enuresis) and urinary retention in one patient. Urinary ultrasounds showed no urinary tract dilatation in any of the patients.

At the last follow-up, three patients reported a complete resolution of urological symptoms. In the other three cases, symptoms were still present, mainly due to low adherence to the prescribed treatments (Table 1).

#### 2.7.2. ICIQ-UI-SF

The ICIQ-UI-SF was administered to all the patients. The mean score was 8.6/21. The minimum score was 0/21, achieved by the three patients who no longer have urological symptoms. The maximum score was 21/21, achieved by one patient (Table 1).

### 2.8. Bowel Dysfunction

#### 2.8.1. Bowel Status

Bowel symptoms were reported by four patients (66.6%). All the patients were initially advised to follow conservative management, with proper eating habits (diet and hydration), correct defecation position, and regular bowel habits. Two patients practiced TAI, one in association with osmotic laxatives (Table 1).

#### 2.8.2. PBMST

The Pediatric Bowel Management Scoring Tool showed that most of the patients had good bowel control (four patients). In one case, the score was 8, corresponding with poor management (Table 1).

### 2.9. Quality of Life

#### Pediatrics Quality of Life Questionnaire

The influence of urological and intestinal disorders on quality of life was investigated using the PedsQL.

The mean score was 62.8/100, where 100 represents a higher score on quality of life. The minimum score was 26.8/100 and the maximum score was 93.4/100.

We also investigated the impact on the physical activities of daily life and on the psychosocial sphere. Regarding physical daily activities, the mean score was 62.5/100; the minimum score was 25/100 and the maximum score was 93.7/100. Regarding the psychosocial impact, the mean score on the PedsQL was 63.3/100; the minimum score was 28.3/100 and the maximum score was 93.3/100 (Table 2).

The scores for all the questionnaires are summarized in Table 2.

## 3. Discussion

Multiple sclerosis has various clinical manifestations, and intestinal dysfunction is common. Recent large-scale studies conducted on patients with multiple sclerosis have confirmed that bowel symptoms are highly prevalent, affecting between 30% and 70% of patients [8,18,19]. Constipation is consistently reported as the most common symptom, with prevalence rates ranging from 44% to over 70% [9,19], whereas fecal incontinence is less frequent but still clinically significant, with estimates ranging from 10% to 30% [19,20]. Bowel dysfunction seems to increase with disease duration and neurological disability, as measured by the Expanded Disability Status Scale (EDSS), although even patients with mild disability may present with significant bowel symptoms [18,21]. These symptoms are often underreported by patients and underrecognized by clinicians, leading to suboptimal management [8]. The incidence of POMS ranges from 0.13 to 0.66 cases per 100,000 children annually [14], but the literature about bowel dysfunction in POMS is poor. As in the adult population, both constipation and fecal incontinence can be found in children, with variability between patients and a significant impact on daily life [12].

In our experience, 50% of patients with LUTSs reported bowel symptoms during their first urological evaluation. All of them were affected by constipation. At our follow-up, the three patients with constipation reported that they still had intestinal symptoms, in one case associated with fecal incontinence. Nevertheless, the PBMST showed a good score: one because of mild constipation, while the other two were due to the good clinical results after TAI administration. However, it is important to emphasize the complexity of patient number 3, whose clinical picture is burdened by additional clinical features that may confuse the impact of multiple sclerosis on the development of neurogenic bowel disease. Indeed, in addition to MS, he also suffers from an anorectal malformation and myelomeningocele (with a secondary tethered cord), conditions that can independently lead to alterations in intestinal function and cause NBD. One patient presented with recent-onset constipation but was not undergoing any treatment. According to the PBMST questionnaire, her bowel dysfunction was classified as moderate. According to this result, a more structured screening using diaries and questionnaires instead of patient/relatives interviews could be useful for providing evidence of the real frequency of bowel dysfunction.

**Figure 1:**
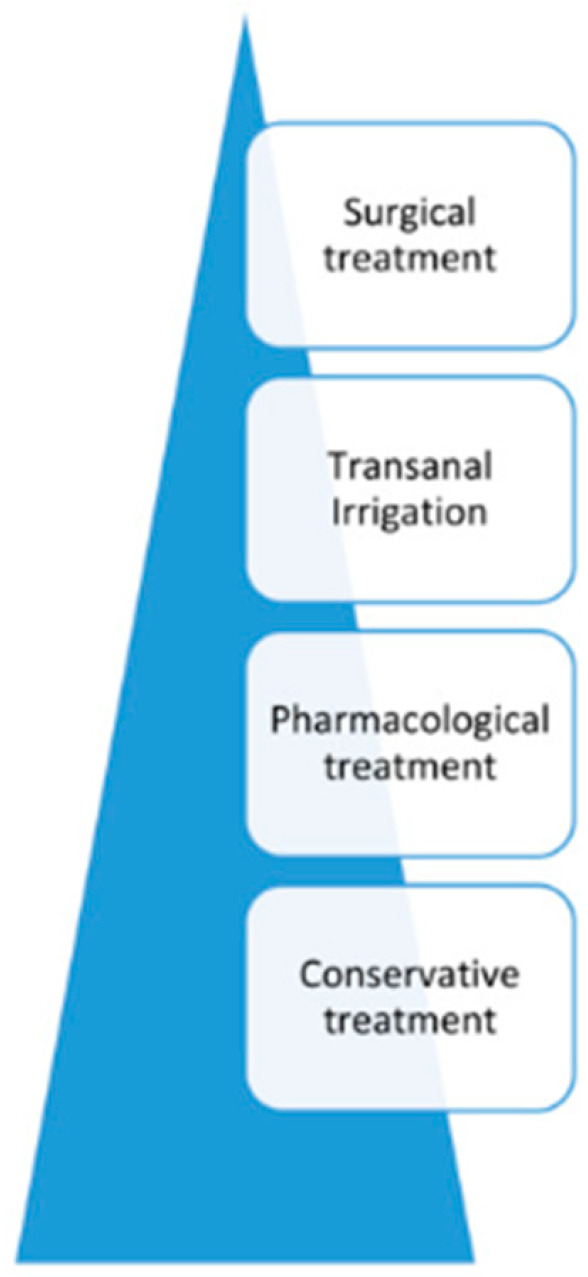
Pyramid of treatment recommendations for neurogenic bowel dysfunction [12].

The “treatment pyramid” (Figure 1) suggests conservative therapy as the first step for bowel management. Conservative therapy begins with simple measures that can be easily integrated into daily life. Modifying the diet to increase fiber intake is typically recommended as an initial step in a bowel management program. The minimal daily fiber requirement (g/day) for children and adolescents (3–20 years) can be estimated using the formula age plus 5 g/day (e.g., 8 g/day at the age of 3 years), while for adults 25–35 g/day is recommended [12]. The efficacy of this diet is correlated with correct fluid intake, as inadequate hydration in combination with increased fiber may exacerbate constipation [12].

Pelvic floor re-education is an effective conservative treatment for bowel dysfunctions both in adults and children, particularly for fecal incontinence and constipation due to dyssynergic defecation [22,23]. This therapeutic approach typically combines pelvic floor muscle training (PFMT), biofeedback, bowel retraining, and, in selected cases, electrical stimulation. Several randomized controlled trials have demonstrated that biofeedback is superior to standard care or pelvic floor exercises alone for reducing the severity and frequency of fecal incontinence episodes [24,25]. Reviews and clinical reports have consistently indicated that PFMT can improve pelvic floor coordination, reduce fecal leakage, and support evacuation in patients with pelvic floor dyssynergia or external anal sphincter weakness [8,26]. The evidence from adult MS cohorts supports biofeedback as an effective intervention for dyssynergic defecation [27,28]. There is no evidence in the literature regarding pelvic floor re-education for the management of fecal dysfunction in children with MS, but studies on neurogenic bowel dysfunction suggest that physiotherapy and biofeedback may be useful in association with lifestyle modifications [8].

Part of pelvic floor rehabilitation involves teaching the correct posture for defecation. The maximum squatting position seems to facilitate the passage of stool, and it is encouraged for neurological bowel dysfunction. The literature suggests that squatting with the hips flexing at 22.5° with respect to the rest of the body increases the anorectal angle to 126° (in normal sitting it is 100°). This position reduces the strain required to pass stool [29].

The important role of physical activity in regulating bowel function is now well known [30]. A recent review suggests that, while moderate activity is a beneficial and well-tolerated intervention for promoting gastrointestinal health, high-intensity activities require careful monitoring, particularly in individuals with pre-existing GI disorders [30]. In the context of MS, the evidence supports the inclusion of structured exercise programs as part of a multimodal management strategy. Aerobic training and pelvic floor exercises have been shown to improve bowel function and continence, likely through enhanced neuromuscular coordination and stimulation of colonic motility [8].

The second step of the “treatment pyramid” (Figure 1) suggest the addition of drugs to conservative management. No specific guidelines for children with multiple sclerosis have been reported. In studies of children affected by NBD, there are several options available. First, there are probiotics. There is currently no specific evidence supporting the use of probiotics in children with neurogenic bowel dysfunction, although they may be considered to promote overall gut health and microbial diversity [12]. Oral laxatives represent the next step in neurogenic bowel dysfunction management, supported by several randomized controlled trials. Polyethylene glycol (PEG) has demonstrated superiority over lactulose in pediatric NBD, producing higher stool frequency [31]. Other agents include stimulant laxatives (bisacodyl, senna), stool softeners (docusate), bulk-forming fibers (ispaghula), and lubricants such as mineral oil. Osmotic laxatives (lactulose, PEG, and milk of magnesia) are generally used to improve hard stool consistency, while stimulants increase bowel frequency. However, dosing must be individualized, as children with NBD often show variable responses and timing of action, which can limit practicality in daily life [32,33]. When oral agents are insufficient, rectal approaches may be employed. Suppositories, such as glycerin, bisacodyl, or newer effervescent sodium bicarbonate preparations, can stimulate evacuation. PEG-based bisacodyl suppositories have shown better efficacy in controlled trials compared to fat-based formulations [34,35,36,37]. Enemas provide another option, particularly when suppositories are ineffective. They may act through mechanical flushing (large-volume solutions) or local stimulation (micro-enemas containing docusate, sodium citrate, or sorbitol). While phosphate enemas can be effective for acute fecal impaction, they are not recommended for routine use due to risks of electrolyte imbalance, dehydration, and mucosal irritation, particularly in children with megacolon or renal compromise [36,37].

In recent years, transanal irrigation has become increasingly popular and successful and represents the third step of bowel management (Figure 1). It is a promising intervention for neurogenic bowel dysfunction in individuals with MS [38]. TAI has been used clinically in children since 1987 for constipation and fecal incontinence, and it is an established option for NBD refractory to conservative and/or pharmacological therapy [39,40]. It is now well known that the regular application of TAI can also reduce fecal incontinence in the long run in up to 90% of patients [11]. A systematic review that included 27 studies and 1.040 pediatric patients (mean age 8 years) reported that 78–84% achieved improved bowel continence and 95% experienced better quality of life following TAI [39]. Two patients in our population reported an increase in their quality of life after starting TAI. TAI is generally well tolerated by patients. Moreover, young adolescents with MS show the high impact of psychological concerns with low adherence to NBD management. For this reason, if conservative therapy is no longer effective, or it is impossible for personal reasons (progressing disease) or social issues, more invasive surgical alternatives can be proposed, as suggested by Figure 1. These include a MACE (Malone Antegrade Continence Enema) stoma [41], where the stoma is usually the appendix or, when unavailable, other intestinal segments (ileum, cecum/colon) [42,43].

We believe that it is extremely important to study the quality of life in chronic patients, particularly among adolescents and young adults. Our case series suggests an association between adherence to urinary and fecal management strategies and higher reported quality of life scores. Lower PedsQL scores were observed in the patients who did not adhere to urological and/or bowel therapy, while the patients undergoing more invasive treatments, such as TAI, reported comparatively higher quality of life scores.

As suggested by the “treatment pyramid” (Figure 1), it is important to use the least invasive approach possible. Likewise, the diagnostic process must also be as non-invasive as possible, and the chance to use a questionnaire is a great option.

In our patients, the associations between the different questionnaires allowed us to better assess their psychological status. In some cases, simultaneous low adherence to bladder and bowel management was present; in others, the patients’ concerns were related to a specific treatment, due to low effectiveness or side effects. We believe these findings can be explained by the chronic nature of the neurological condition, which requires ongoing treatment and frequent hospitalizations, often leading to a progressive refusal of additional therapies. Psychological comorbidities are still scantly investigated in pediatric urology and surgery clinics; these can have an enormous impact on the acceptance of the proposed therapies and, therefore, on the clinical outcome.

Limits of the Study: The small sample size, as this is a single-center case series, did not allow for a significant statistical analysis. Furthermore, the population analyzed consists of patients followed by our unit for the onset of urological symptoms, and does not include patients who may have only presented with neurogenic bowel due to MS. The patient group in this study is also heterogeneous, including one patient with significant comorbidities (previous anorectal malformation and spina bifida), which may have further worsened the neurogenic bladder and bowel symptoms induced by multiple sclerosis.

Moreover, the assessment of QoL using the PedsQL questionnaire represents a patient-reported perception and does not include an objective psychosocial assessment. Furthermore, the adherence to therapy and the patient’s psychological status were not assessed using questionnaires, but were subjectively evaluated by the examiners/clinicians based on the questionnaires utilized and the clinical follow-up.

## 4. Conclusions

The incidence of multiple sclerosis in the pediatric population is steadily increasing, and with it its various symptomatic manifestations. Urological and fecal symptoms do not play a central role among the clinical manifestations of multiple sclerosis, but they may already be present in adolescence, burdening the quality of life. The proper diagnosis and treatment of these symptoms appear to lead to higher scores on QoL assessments in young patients. Questionnaires appear to be extremely useful tools for researching these symptoms and identifying their severity, with the advantage of not being invasive instruments.

This work was generated within the European Reference Network for Rare Urogenital Diseases and Complex Conditions (ERN EUROGEN).

## Figures and Tables

**Table 2:** Scores collected from all questionnaires.

Pt No; Sex	ICIQ-UI-SF Score (0–21)	PBMST	PedsQL Physical Psychosocial Score (Tot: 100)	PedsQL Physical Activity Score (Tot: 100)	PedsQL Total Score (Tot: 100)
1; female	19	2 (fair)	28.3	25	26.08
2; female	12	2 (fair)	53.3	43.7	50
3; male	21	6 (moderate)	70	90.6	77.2
4; female	0	1 (fair)	93.3	93.7	93.5
5; female	0	8 (poor)	53.3	37.0	47.8
6; male	0	0 (fair)	81.6	84.4	82.6

## Data Availability

The data presented in this study are available on request from the corresponding author due to ethical reasons.

## Abbreviations

The following abbreviations are used in this manuscript:

MS	Multiple Sclerosis
CNS	Central Nervous System
QoL	Quality of Life
POMS	Pediatric-Onset Multiple Sclerosis
EOMS	Early-Onset MS
NBD	Neurogenic Bowel Dysfunction
ICIQ-UI SF	International Consultation on Incontinence Questionnaire-Urinary Incontinence Short Form
PBMST	Pediatric Bowel Management Scoring Tool
PedsQL	Pediatric Quality of Life
LUTSs	Lower Urinary Tract Symptoms
CIC	Clean Intermitted Catheterization
TAI	Transanal Irrigation
RR	Relapsing–Remitting
RR-MS	Relapsing–Remitting Multiple Sclerosis
EDSS	Expanded Disability Status Scale
PFMT	Pelvic Floor Muscle Training
GI	Gastrointestinal
PEG	Polyethylene Glycol
MACE	Malone Antegrade Continence Enema
